# Research progress of potassium-competitive acid blockers in the treatment of *Helicobacter pylori*

**DOI:** 10.1080/07853890.2025.2544886

**Published:** 2025-08-12

**Authors:** Zhaoli Ma, Shiling Wang, Furui Wang, Qian Ren

**Affiliations:** ^a^ The First School of Clinical Medicine, Lanzhou University, Lanzhou, Gansu Province, China; ^b^ Department of Gastroenterology, The First Hospital of Lanzhou University, Lanzhou, Gansu Province, China; ^c^ Gansu Health Vocational College, Lanzhou, Gansu Province, China; ^d^ Gansu Province Clinical Research Center for Digestive Diseases, Lanzhou University, Lanzhou, Gansu Province, China

**Keywords:** Eradication therapy, gut microbiota, *Helicobacter pylori*, potassium-competitive acid blocker

## Abstract

*Helicobacter pylori* (*H. pylori*) infection is a primary pathogen responsible for gastrointestinal diseases, including gastritis, peptic ulcer disease, and gastric cancer. Therefore, the eradication of *H. pylori* is crucial for the prevention and treatment of these conditions. However, the increasing antibiotic resistance has led to a gradual decline in the efficacy of traditional Proton Pump Inhibitors (PPIs) in triple or quadruple therapy, posing new challenges for *H. pylori* eradication. Potassium-competitive acid blockers (P-CAB) represent a novel class of acid-suppressing drugs that exert potent and sustained acid suppression by competitively inhibiting the potassium-binding site of the H^+^/K^+^-ATPase. Compared to traditional PPI, P-CAB offers advantages such as rapid onset of action, prolonged duration of effect, and less susceptibility to the influence of CYP2C19 genetic polymorphism, highlighting their significant potential in *H. pylori* eradication therapy. This article aims to comprehensively review the clinical efficacy and safety of P-CAB-containing regimens in first-line and rescue treatments for *H. pylori* eradication. This includes but is not limited to dual therapy combining P-CAB with other antibacterial agents, triple therapy, and quadruple therapy in combination with bismuth. By analyzing existing clinical research data, we will explore the application effects of P-CAB in different treatment regimens, as well as the advantages and potential limitations compared with PPI. Additionally, this article will focus on the tolerability of these treatment regimens in patients and the incidence of adverse reactions, to provide scientific evidence and new perspectives for clinicians in selecting appropriate *H. pylori* eradication regimens.

## Introduction

*Helicobacter pylori* (*H. pylori)* is a significant pathogen responsible for gastritis, peptic ulcer disease, gastric adenocarcinoma, mucosa-associated lymphoid tissue lymphoma, and gastric mucosal-related ulcers [1,2]. Therefore, the Kyoto Global Consensus Report, the Sixth Chinese National Consensus Report, and the Maastricht VI/Florence Consensus Report all recommend the eradication of *H. pylori* infection [3–5]. Currently, the global efficacy of the standard triple therapy for *H. pylori* treatment has dropped below 80% [6,7]. The main reasons for eradication failure include antibiotic resistance, poor compliance, reduced gastric pH, high bacterial load, and the rapid metabolism of proton pump inhibitors (PPIs) in the human body. Previous reports have shown that the resistance rates to clarithromycin, amoxicillin, metronidazole, tetracycline, and levofloxacin are 56–71%, 0–8%, 35–74%, 0–4%, and 21–43%, respectively [5,8–12]. Common eradication regimens include the combination of PPI with two different antibiotics, with or without bismuth. Although these regimens initially achieved a 90% cure rate, the success rate of eradication has gradually declined due to increased antibiotic resistance and insufficient acid suppression [13]. In response to current clinical needs, a novel potassium-competitive acid blocker (P-CAB) has emerged. As a new generation of highly efficient and selective gastric H^+^/K^+^-ATPase acid suppressant, it has potent acid suppression and the ability to overcome the limitations of PPIs. This breakthrough provides a new option for *H. pylori* eradication therapy, potentially significantly enhancing the eradication rate. Therefore, this article aims to systematically summarize the efficacy and safety of first-line and second-line treatment regimens based on P-CAB in eradicating *H. pylori*, providing a scientific and practical reference for clinical treatment.

## Overview of P-CAB

### Representative P-CAB

Currently, the clinically prominent novel P-CABs include Vonoprazan (VPZ), Tegoprazan (TPZ), and Keverprazan (KPZ) [14]. Among these, VPZ was first launched in Japan in December 2014 and has been approved for various indications, such as reflux esophagitis, gastric ulcer, duodenal ulcer, prevention of recurrence of gastric or duodenal ulcers, and eradication of *H. pylori* [15,16]. Subsequently, VPZ received approval for marketing in China in December 2019, primarily for the treatment of reflux esophagitis [17]. In October 2021, VPZ obtained a new indication in China, which is to be used in combination with appropriate antibiotics for the eradication of *H. pylori* [4]. TPZ, approved in South Korea in July 2018, is the first P-CAB used clinically for the treatment of non-erosive reflux disease and was later launched in China in 2022 [18]. KPZ, approved in China in February 2023, is the first P-CAB to have dual indications for the treatment of both duodenal ulcer and reflux esophagitis post-marketing [19].

### Mechanism of action of P-CAB

As a new generation of highly efficient and selective gastric H^+^/K^+^-ATPase acid suppressants, P-CAB exhibit numerous unique advantages: (1) They do not require activation in a strongly acidic environment, can simultaneously inhibit both resting and activated H^+^/K^+^-ATPase, takes effect from the first dose, works immediately, and has a long duration of acid suppression [20]; (2) They offer flexible dosing timing, being effective whether taken on an empty stomach or after meals, enhancing the convenience of medication [21]; (3) They are primarily metabolized by CYP3A4 and are not affected by CYP2C19 genetic polymorphism, ensuring stable efficacy across different genetic backgrounds [22].

### Comparison between P-CAB and PPI

P-CAB competes directly with the potassium ion channel on the proton pump, eliminating the need for conversion to an active form in an acidic environment, thereby acting faster and significantly inhibiting gastric acid secretion within hours. In contrast, PPIs are unstable in acidic environments and require conversion to their active form under specific conditions to exert their effect, typically taking 2–3 days to achieve maximum acid suppression [23]. In recent years, studies have confirmed that P-CAB is more potent and has a longer-lasting effect than PPI in inhibiting gastric acid secretion [24]. A study shows that the half-life of VPZ is 6 to 8 h, and it does not require repeated administration, whereas PPI requires frequent and high-dose administration and is prone to inducing or exacerbating atrophic gastritis caused by *H. pylori* infection. If P-CAB drugs are used instead of PPI, it will be beneficial to reduce the risk of its occurrence [25,26]. Additionally, a study involving healthy volunteers demonstrated that, compared to PPI, VPZ achieved a higher, faster, and more sustained increase in intragastric pH, with virtually no occurrences of nocturnal acid breakthrough [27–29]. Furthermore, a small-sample randomized clinical trial evaluated the pharmacodynamics following administration of different doses of TPZ or pantoprazole combined with amoxicillin or clarithromycin. The results showed that, on the first day of treatment, the intragastric pH in the TPZ group reached above 6 and remained at this level for 24 h. Both the median pH and the percentage of time with pH >4 or pH >6 were higher in the TPZ group compared to the PPI group [30]. Similarly, the results of a controlled study showed that KPZ can rapidly, significantly, and sustainably suppress gastric acid secretion. Within 24 h, the proportion of time with an intragastric pH > 5 reached over 80%, and even exceeded 95% during the night. Furthermore, the half life of KPZ is 6.00–7.17 h, whereas that of lansoprazole is only 2 h. The acid-suppressing effect of KPZ is superior to that of PPI [31]. And compared to PPI, relevant studies have pointed out that VPZ, TPZ, and KPZ are not affected by food intake, offering patients better convenience in taking the medication [31–33]. Additionally, PPIs are primarily metabolized in the liver *via* the CYP2C19 enzyme. Patients with the rapid-metabolizer CYP2C19 genotype exhibit poorer acid-suppressing effects. But P-CAB, which mainly relies on CYP3A4 for metabolism, can effectively avoid individual differences [22]. Overall, we recommend using P-CABs in *H. pylori* treatment. The Pharmacokinetics (PK) and Pharmacodynamics (PD) of VPZ, TPZ, and KPZ are presented in Table 1 [34–36].

**Table 1.** PK and PD characteristics of currently approved VPZ, TPZ, and KPZ (at approved doses) in healthy Asian subjects under fasting conditions in the morning.

Medicine (Dosage)	Vonoprazan (20mg qd)	Tegoprazan (50mg qd)	Keverprazan (20mg qd)
Molecular formula	C_17_H_16_FN_3_O_2_S	C_20_H_19_F_2_N_3_O_3_	C_22_H_25_FN_2_O_4_S
*p*Ka	9.06	5.10	9.12
*T*_max_ (h)	1.5 (1.5 − 3.0)	1.0 (0.5 − 2.0)	2.0 (1.2 − 2.0)
Half life (h)	6.9 ± 1.6	4.1 ± 1.4	6.3 ± 1.2
24h pH, U (Day 1)	NA	4.1 ± 0.7	NA
24h pH, U (Day 7)	NA	4.7 ± 1.1	NA
24h pH >4 HTR % (Day 1)	63.3 ± 17.9	54.5 ± 17.9	85.0 ± 3.0
24h pH >4 HTR % (Day 7)	83.4 ± 16.7	68.2 ± 24.9	98.3 ± 4.3

*p*Ka: Acid dissociation constant; *T*max: Time to maximum plasma concentration; Half-life: Elimination half-life; HTR: Holding time ratios; qd: Quaque die; NA: Not available.

## Dual therapy based on P-CAB as first-line treatment

The Maastricht VI/Florence Consensus Report highlights that the efficacy of the dual regimen, which combines VPZ and amoxicillin, in eradicating *H. pylori* surpasses that of the conventional PPI-based triple therapy [5].

### VPZ-based dual therapy

Research reports have shown that the dual therapy of VPZ combined with amoxicillin (VA) exhibits superior efficacy when compared to the dual therapy of esomeprazole combined with amoxicillin (EA). The VA therapy group achieved a satisfactory eradication rate (≥90%), with effects not inferior to those of the EA dual therapy [37]. A multicenter randomized clinical study from China made an in-depth comparison between VA dual therapy and the esomeprazole quadruple therapy (EBAM) in terms of clinical efficacy and safety. The results revealed that the VA group was comparable to the EBAM group in terms of *H. pylori* eradication rate, and had a significantly lower incidence of adverse reactions (16.67% vs. 38.04%, *p* < 0.01) [38]. A retrospective meta-analysis explored the efficacy of VA dual therapy in *H. pylori* treatment. The results showed that the eradication rate of VA therapy (88.5%) was significantly superior to that of the triple therapy based on VPZ (*p* < 0.01). The study further conducted a subgroup analysis of clarithromycin-sensitive and resistant strains, finding that for clarithromycin-sensitive strains, the eradication rate of the VPZ dual therapy was slightly lower than that of the VPZ-based triple therapy. In contrast, for clarithromycin-resistant strains, the dual therapy was significantly superior to the triple therapy (86.7% vs. 71.4%, *p* < 0.05) [39]. In 2023, a randomized clinical study conducted in China explored the efficacy of the 10-day V–A dual therapy in eradicating *H. pylori*. The results were encouraging, showing that this therapy achieved a satisfactory eradication rate of ≥90% [40]. Meanwhile, another multicenter randomized clinical study conducted in China compared the clinical efficacy and safety of the 10-day VA dual therapy with the rabeprazole combined with amoxicillin dual therapy (RA). The study results showed that the VA group significantly outperformed the RA group regarding *H. pylori* eradication rate (91.4% vs. 86.6%, *p* < 0.05), with a lower incidence of adverse reactions [41]. Conversely, previous studies have shown that the 10-day PPI dual therapy results were not ideal (≤80%) [42,43]. But the satisfactory eradication rates achieved in studies may be related to the strong and sustained gastric acid suppression capability of VPZ. Overall, the 10-day and 14-day VA dual therapies have demonstrated satisfactory eradication rates for *H. pylori*. While the 10-day regimen offers advantages in terms of shorter treatment duration, lower incidence of adverse reactions, and reduced medical costs compared to the traditional 14-day therapy, the 14-day regimen may be more suitable for regions with high antibiotic resistance rates or patients with a history of treatment failure. Regarding VPZ-based dual therapy, in addition to a large number of multicenter RCTs, there is also considerable evidence from meta-analysis. Several meta-analysis results support the view that VPZ combined with amoxicillin therapy has a high eradication rate, low adverse reactions, good compliance, and is a highly potent first-choice regimen [44–46]. Compared with Asian populations, the VPZ dual therapy yields less satisfactory eradication rates in European and American countries [47]. Antibiotic resistance was first considered, but a US study (2018–2019) found only 1.1% *H. pylori* resistance to amoxicillin [48]. Therefore, we think the differing eradication rates across studies are probably linked to regional differences. Data indicates that there is a higher prevalence of overweight and obesity in European and American countries [49]. And research indicates that an increase in Body Surface Area (BSA) and Body Mass Index (BMI) is associated with suboptimal eradication rates for VPZ dual therapy [50,51]. Increased body fat in obese patients can expand the blood’s antibiotic distribution volume, risking drug levels too low to effectively kill bacteria [52,53]. We speculate that changes in drug distribution, metabolism, and excretion may affect the pharmacokinetics of VPZ, thereby reducing its therapeutic effect. We also noted that antibiotic and VPZ administration timing differs (pre-meal in Western countries, post-meal in Japan). This difference could potentially affect gastric antibiotic exposure, influencing eradication efficacy [24]. Therefore, we think future studies in European and American regions need to optimize VA dual therapy’s dosage and duration, and assess its efficacy and safety.

Encouragingly, two recent studies have proposed a novel treatment regimen based on VPZ, which combines VPZ with tetracycline [54,55]. These studies consistently showed that for the special population allergic to penicillin, this regimen can achieve a satisfactory eradication rate (≥90%) with good safety. The suitability of VPZ and tetracycline for non-penicillin-allergic *H. pylori* patients requires further study to confirm efficacy and safety.

### TPZ-based dual therapy

The latest multicenter, randomized clinical study conducted in China in 2024 compared the clinical efficacy and safety of a 14-day TPZ-based dual therapy (including TPZ and amoxicillin, TA) with a PPI-based bismuth quadruple therapy. The results demonstrated that the TA dual therapy had a significantly higher eradication rate than the PPI-based quadruple therapy, with no adverse events observed during treatment [56]. And another multicenter, non-inferiority, randomized controlled clinical trial compared the efficacy and safety of TA dual therapy with esomeprazole–amoxicillin dual therapy (EA) for *H. pylori* infection. The results showed that the eradication rates of both groups were comparable, but the incidence of adverse reactions in the TA group was significantly lower than that in the EA group [57]. Additionally, a multicenter randomized controlled study compared the performance of TA dual therapy with TPZ-based quadruple therapy in the treatment of *H. pylori* infection. The results indicated that the eradication rate of the TA dual therapy was non-inferior to that of the TPZ quadruple therapy, and the incidence of adverse reactions in the TA group was significantly lower than that in the TPZ quadruple therapy group [58]. Although the eradication rates in the aforementioned studies were slightly below the ideal target of 90% [57,59], they can still be considered acceptable. We speculate that the resistance of *H. pylori* to amoxicillin may vary by region and population, and high resistance rates may affect the eradication rate. Overall, TA dual therapy shows potential advantages in the eradication treatment of *H. pylori* infection.

In summary, the treatment regimen using P-CAB as acid suppression agents has demonstrated high efficacy and safety in the eradication of *H. pylori*. However, currently, there is relatively limited research on P-CAB-based dual therapy, and the existing clinical data are not sufficient to fully support this theory. Future research will require large-scale, multicenter clinical studies to confirm its effectiveness and safety (Table 2).

**Table 2.** Comparison of *H. pylori* eradication rate and safety based on P-CAB dual therapy regimen.

First author	Year of publication	Country	Study design	Number of patients enrolled	Experimental design	Eradication rate	Incidence rate of adverse reactions	Confirmative test for eradication
Su et al. [37]	2023	China	Retrospective cohort study	213	VA: VPZ 20 mg bid + AMX 1 g tid, 14d EA: EPZ 20 mg qid + AMX 750 mg qid, 14d	ITT: 89.0% vs.87.7% PP: 94.1% vs. 92.8%	16.4% vs. 8.3%	UBT
Hu et al. [38]	2023	China	Randomized controlled trial	194	VA: VPZ 20 mg bid + AMX 1 g tid, 14d EBAM: EPZ 20 mg bid + BPC 220 mg bid + AMX 1 g bid + MTZ 0.4 g qid, 14d	ITT: 88.7% vs. 91.8% PP: 95.6% vs. 96.7%	16.67% vs. 38.04%	UBT
Qian et al. [40]	2023	China	Randomized controlled trial	375	H-VA: VPZ 20 mg bid + AMX 750 mg qid, 10d L-VA: VPZ 20 mg bid + AMX 1000 mg bid, 10d EBAC: EPZ 20 mg bid + BPC 200 mg bid + AMX 1000 mg bid + CLR 500 mg bid, 10d	ITT: 91.2% vs. 82.4% vs. 88.0% PP: 93.4% vs. 85.1% vs. 90.9%	11.38% vs. 8.2% vs. 23.58%	UBT
Han et al. [41]	2023	China	Randomized controlled trial	600	VA: VPZ 20 mg bid + AMX 1 g tid, 10d RA: RPZ 10 mg tid + AMX 1 g tid, 14d	ITT: 89.3% vs. 84.9% PP: 91.4% vs. 86.6%	8.4% vs. 9.0%	UBT
Gao et al. [54]	2023	China	Retrospective cohort study	62	VT: VPZ 20 mg bid + TET 500 mg tid (body weight < 70 kg) or 500 mg qid (body weight ≥ 70 kg), 14d	Overall: 93.5% First-line treatment group: 100% Rescue treatment group: 90.9%	Overall: 9.7% First-line treatment group: 27.8% Rescue treatment group: 4.5%	UBT
Gao et al. [55]	2024	China	Randomized controlled trial	300	VT: VPZ 20 mg bid + TET 500 mg tid, 14d LBTM: LPZ 30 mg bid + CB 150 mg tid + TET 500 mg tid + MTZ 400 mg tid, 14d	ITT: 92.0% vs. 89.3% PP: 95.1% vs. 97.7%	14.0% vs. 48%	UBT
Lin et al. [56]	2024	China	Randomized controlled trial	321	TZ: TPZ 50 mg qd + AMX 1 g tid, 14d TZ: TPZ 50 mg bid + AMX 1 g bid, 10d EBAC: EPZ 20 mg bid + BPC 240 mg bid + AMX 1 g bid + CLR 500 mg bid, 14d	ITT: 85.98% vs. 85.98% vs. 85.05% PP: 91.00% vs. 93.81% vs. 90.81%	13.33% vs. 14.56% vs. 27.18%	UBT
Kong et al. [57]	2024	China	Randomized controlled trial	368	TA: TPZ 50 mg bid + AMX 750 mg qid, 14d EA: EPZ 20 mg qid + AMX 750 mg qid, 14d	ITT: 85.8% vs. 84.2% PP:88.2 vs. 88.5%	16.3% vs. 21.2%	UBT
Liu et al. [59]	2024	China	Randomized controlled trial	236	TA: TPZ 50 mg bid + AMX 1 g tid, 14d TBAC: TPZ 50 mg bid + BPC 0.6 g bid + AMX 1 g bid + CLR 0.5 g bid, 14d	ITT: 83.9% vs. 81.4% PP: 88.3% vs. 84.8%	4.2% vs. 15.3%	UBT

VPZ, Vonoprazan; TPZ, Tegoprazan; KPZ, Keverprazan; EPZ, Esomeprazole; RPZ, Rabeprazole; LPZ, Lansoprazole; AMX, Amoxicillin; MTZ, Metronidazole; CLR, Clarithromycin; TET, Tetracycline; BPC, Bismuth potassium citrate; ITT, Intention-to-treat; PP, Per-protocol; UBT, Urea breath test; qd, Quaque die; bid, Bis in die; tid, Ter in die; qid, Quater in die.

## Triple therapy based on P-CAB as first-line treatment

In Japan, the VPZ-based triple therapy has been recommended for the treatment of *H. pylori* infection [60]. And in 2022, the VPZ-based triple therapy was also approved in the United States for the treatment of *H. pylori* infection [47].

### VPZ-based triple therapy

In a meta-analysis focusing on the efficacy of VPZ in eradicating *H. pylori*, researchers comprehensively compared the effectiveness of various treatment regimens in *H. pylori* eradication [14]. This analysis included data from 15 studies, encompassing a total of 8049 patients. The results demonstrated that the VPZ-based triple therapy (consisting of VPZ, amoxicillin, and clarithromycin) was significantly superior in eradication rates compared to the PPI-based triple therapy, with a statistically significant difference (*p <* 0.001). In a prospective randomized clinical study, 641 patients with initial *H. pylori* infection were enrolled [61]. The study found that the eradication rate of *H. pylori* with the VPZ triple therapy (92.6%) was significantly higher than that of the PPI triple therapy (75.9%), with a difference of 16.7%. This result not only confirmed the non-inferiority of VPZ but also demonstrated its statistically significant superiority (*p <* 0.05).

The results of a Phase III clinical trial conducted in the United States and Europe showed that the VPZ triple therapy was non-inferior to the PPI triple therapy in eradicating *H. pylori*, with significant effectiveness. Further subgroup analysis revealed that even in the face of clarithromycin-resistant strains, the VPZ triple therapy maintained a higher eradication rate, showing a clear advantage over the lansoprazole-based triple therapy [47]. We believe that VPZ has the potential to overcome clarithromycin resistance, thereby enhancing treatment efficacy, a finding consistent with previous research [62]. A randomized controlled trial compared the clinical efficacy of the V-AC therapy (including VPZ, amoxicillin, clarithromycin) with the S-14 therapy (lansoprazole and amoxicillin for the first 7 days, followed by lansoprazole, clarithromycin, and metronidazole for the next 7 days) [63]. The results showed that the eradication rates of the VAC group and the S-14 group were comparable (88.6% vs. 90.3%). Although the VAC regimen did not achieve an eradication rate of over 90% in this study, it still met the non-inferiority standard. It is noteworthy that the VAC regimen in this study was implemented for only 7 days, highlighting its advantages of short treatment duration, low drug burden, high safety, and good compliance. Overall, VPZ provides a strong alternative for *H. pylori* eradication treatment.

### TPZ-based triple therapy

Previous studies on healthy subjects have shown that when TPZ is combined with amoxicillin or clarithromycin, the exposure to TPZ significantly increases, which can substantially enhance the pharmacological response [30]. In a Retrospective cohort study, researchers administered TPZ-based triple therapy to 948 patients for treatment durations of 7 and 14 days. The results showed that the eradication rate in patients who received the 14-day TPZ triple therapy was significantly higher than that in the 7-day treatment group (85.1% vs. 70.5%, *p* < 0.001), with this difference being statistically significant [64]. However, in a clinical trial, researchers compared the clinical efficacy of a 7-day TPZ triple therapy with a PPI-based triple therapy in eradicating *H. pylori*, and the results did not meet expectations, with an eradication rate of only 69.33% [65]. In this study, the TPZ group did not demonstrate superiority over the PPI group, showing only non-inferiority. Further subgroup analysis identified clarithromycin resistance as the only significant risk factor for eradication failure. Although both TPZ and VPZ belong to the P-CAB class of drugs and are theoretically expected to have similar clinical efficacy, existing research results do not support this. We speculate that this may be due to TPZ’s short half-life, differences in binding sites with H^+^/K^+^-ATPase, as well as the chemical structural similarity (benzimidazole structure) between TPZ and PPIs, antibiotic resistance rates, and variations in the Minimum Inhibitory Concentration (MIC) distribution of *H. pylori* to clarithromycin and amoxicillin [66–69]. These factors may reasonably explain why the eradication rate of TPZ is lower than that reported in Japanese studies. Therefore, when selecting a treatment regimen, it is important to consider local antibiotic resistance rates and individual patient factors for personalized decision-making.

Overall, both VPZ and TPZ, as P-CAB agents, have demonstrated high eradication rates and favorable safety profiles in the triple therapy for *H. pylori* eradication. Therefore, in clinical practice, the P-CAB-based triple therapy is recommended as the preferred treatment option for *H. pylori* infection (Table 3).

**Table 3.** Comparison of *H. pylori* eradication rate and safety based on P-CAB triple therapy regimen.

First author	Year of publication	Country	Study design	Number of patients enrolled	Experimental design	Eradication rate	Incidence rate of adverse reactions	Confirmative test for eradication
Murakami et al. [61]	2016	Japan	Randomized controlled trial	650	First-line therapy: VAC: VPZ 20 mg bid + AMX 750 mg bid + CLR 200/400 mg bid, 7d LAC: LPZ 30 mg bid + AMX 750 mg bid + CLR 200/400 mg bid, 7d Second-line therapy: VAM: VPZ 20 mg bid + AMX 750 mg bid + MTZ 250 mg bid, 7d	First-line therapy: ITT: 92.6% vs. 75.9% PP: 92.6% vs. 75.9% Second-line therapy: ITT: 98% PP: 98%	First-line therapy: 34.0% vs. 41.1% Second-line therapy: 30.0%	UBT
Chey et al. [47]	2022	US & Europe	Randomized controlled trial	1046	VA: VPZ 20 mg bid + AMX 1 g tid, 14d VAC: VPZ 20 mg bid + AMX 1 g bid + CLR 500 mg bid, 14d LAC: LPZ 30 mg bid + AMX 1 g bid + CLR 500 mg bid, 14d	ITT: 78.5% vs. 84.7% vs. 78.8% PP: 77.2% vs. 80.8% vs. 68.5%	34.1% vs. 34.1% vs. 34.5%	UBT
Chiu et al. [63]	2024	Taiwan, China	Randomized controlled trial	628	VAV: VPZ 20 mg bid + AMX 1 g bid + CLR 500 mg bid, 7d S-14: first 7 days, LPZ 30 mg bid + AMX 1 g bid; next 7 days, LPZ 30 mg bid + CLR 500 mg bid + MTZ 500 mg tid	ITT: 81.8% vs. 81.4% PP: 88.6% vs. 90.3%	Overall adverse events (AEs) rate: 74.3% vs. 72.1% severe AEs rate: 14.5% vs. 23.1%	UBT
Jung et al. [64]	2022	South Korea	Retrospective cohort study	948	VAC-7d: TPZ 50 mg bid + AMX 1 g bid + CLR 500 mg bid, 7d VAC-14d: TPZ 50 mg bid + AMX 1 g bid + CLR 500 mg bid, 14d	ITT: 63.9% vs. 78.6% PP: 70.5% vs. 85.1%	26.4% vs. 29.2%	UBT
Chio et al. [65]	2022	South Korea	Randomized controlled trial	350	TAC: TPZ 50 mg bid + AMX 500 mg bid + CLR 500 mg bid, 7d LAC: LPZ 30 mg bid + AMX 500 mg bid + CLR 500 mg bid, 7d	ITT: 62.86% vs. 60.57% PP: 69.33% vs. 67.33%	41.86% vs. 39.31%	UBT

VPZ, Vonoprazan; TPZ, Tegoprazan; LPZ, Lansoprazole; AMX, Amoxicillin; MTZ, Metronidazole; CLR, Clarithromycin; TET, Tetracycline; ITT, Intention-to-treat; PP, Per-protocol; UBT, Ureabreath test; bid, Bis in die; tid, Ter in die.

## Quadruple therapy based on P-CAB as first-line treatment

Accordingly, both the Kyoto Global Consensus and the 2022 Chinese Guidelines for the Treatment of *H. pylori* Infection recommend the bismuth quadruple regimen containing P-CAB as a treatment option for *H. pylori* eradication [3,4].

### VPZ-based quadruple therapy

Although current research on VPZ-based quadruple therapy is relatively limited and clinical evidence is still insufficient, a single-center randomized clinical study conducted in China has provided new insights into this field [70]. This study compared the efficacy of VPZ bismuth quadruple therapy with PPI in the eradication of *H. pylori*, showing that VPZ in quadruple therapy is as effective as PPI, with similar eradication rates for VPZ-10d and VPZ-14d, and offering a more cost-effective option. Furthermore, a multicenter randomized clinical study conducted across 19 regions in China enrolled 510 patients, including those undergoing initial treatment and those who had previously failed PPI-based eradication. These patients were randomly assigned to a 14-day VPZ quadruple group or an esomeprazole quadruple group. The results indicated that the eradication rates of *H. pylori* were comparable between the VPZ and EPZ groups (87.4% vs. 86.3%, *p* < 0.001), with good tolerability of VPZ, not inferior to EPZ [71]. Although the eradication rates in both groups did not reach 90%, we analyzed that this might be related to some patients’ previous exposure to PPI and antibiotic therapy, potentially leading to antibiotic resistance. Additionally, a recent study from Korea highlighted that VPZ quadruple therapy is safe and well-tolerated for *H. pylori* eradication, with efficacy comparable to PPI regimens [72]. However, the study was limited by a small sample size (only 26 cases), and its conclusions require further validation in larger populations to fully assess the effectiveness of VPZ-containing quadruple therapy. Nonetheless, VPZ quadruple therapy remains a promising alternative for the treatment of *H. pylori* infection, especially in cases of clarithromycin resistance.

### TPZ-based quadruple therapy

A randomized, double-blind, active-controlled trial thoroughly compared the TPZ-based bismuth quadruple therapy (consisting of TPZ, tetracycline, metronidazole, and bismuth) with the PPI-based bismuth quadruple therapy (consisting of LPZ, tetracycline, metronidazole, and bismuth). The results showed that the TPZ quadruple group was non-inferior to the PPI group in terms of *H. pylori* eradication rate, with rates of 90.2% and 82.4% respectively (*p* < 0.001), and both groups exhibited similar safety profiles [73]. A retrospective study conducted in South Korea compared the clinical efficacy of TPZ quadruple therapy with TPZ triple therapy for first-line treatment of *H. pylori* infection. The results demonstrated a significant advantage of TPZ quadruple therapy in eradicating *H. pylori*, with an eradication rate of 95.8%, which was significantly higher than the 87.5% achieved by TPZ triple therapy (*p* < 0.05) [74]. Additionally, a single-arm randomized clinical study enrolled 84 patients and administered a 10-day TPZ quadruple therapy, which resulted in a high eradication rate of 96.2% and showed good safety [75]. Furthermore, antibiotic susceptibility testing was performed on 73 of these patients, revealing that 26.0% were resistant to clarithromycin, 42.5% to metronidazole, and 11.0% to both clarithromycin and metronidazole. Notably, despite the presence of antibiotic resistance, 66.1% of the patients successfully eradicated *H. pylori* after treatment, indicating the potential of TPZ to combat antibiotic resistance and thereby enhance eradication rates. Therefore, TPZ is a promising alternative for the eradication of *H. pylori*.

### KPZ-based quadruple therapy

A multicenter randomized clinical study conducted in China enrolled 576 patients [76], who were administered either a 14-day KPZ quadruple regimen or an esomeprazole quadruple regimen. The results demonstrated that the KPZ quadruple group achieved a significantly higher *H. pylori* eradication rate compared to the EPZ group (93.49% vs. 88.24%, *p* < 0.05). Furthermore, subgroup analyses were performed for clarithromycin-resistant and non-resistant patients, revealing that the KPZ group had higher eradication rates in both subgroups. We speculate that the KPZ-based quadruple therapy may be more effective against clarithromycin-resistant *H. pylori* strains. Therefore, KPZ is a promising candidate for effective *H. pylori* eradication treatment. Although clinical research on KPZ is currently limited, the preliminary results of the KPZ quadruple therapy are encouraging.

The aforementioned research findings indicate that the P-CAB-based bismuth quadruple therapy is comparable in efficacy to the PPI-based bismuth quadruple therapy for the eradication of *H. pylori*, and may even surpass it in effectiveness. Based on these findings, we advocate that the P-CAB-based bismuth quadruple therapy can be considered a viable option for the treatment of *H. pylori* infection (Table 4).

**Table 4.** Comparison of *H. pylori* eradication rate and safety based on P-CAB quadruple therapy regimen.

First author	Year of publication	Country	Study design	Number of patients enrolled	Experimental design	Eradication rate	Incidence rate of adverse reactions	Confirmative test for eradication
Lu et al. [70]	2023	China	Randomized controlled trial	234	VAFB-10d: VPZ 20 mg bid + AMX 1 g bid + FZD 100 mg bid + CBS 200 mg bid, 10d VAFB-14d: VPZ 20 mg bid + AMX 1 g bid + FZD 100 mg bid + CBS 200 mg bid, 14d EAFB: EPZ 20 mg bid + AMX 1 g bid + FZD 100 mg bid + CBS 200 mg bid, 14d	ITT: 96.2% vs. 94.9% vs. 93.6% PP: 98.6% vs. 97.4% vs. 94.8%	12.8% vs. 3.8% vs. 6.4%	UBT
Song et al. [71]	2025	China	Randomized controlled trial	510	VBAC: VPZ 20 mg bid + BPC 600 mg bid + AMX 1 g bid + CLR 500 mg bid, 14d EBAC: EPZ 20 mg bid + BPC 600 mg bid + AMX 1 g bid + CLR 500 mg bid, 14d	ITT: 86.8% vs. 86.7% PP: 87.4% vs. 86.3%	69.1% vs. 61.4%	UBT
Kim et al. [73]	2023	South Korea	Randomized controlled trial	217	TBMT: TPZ 50 mg bid + CBS 300 mg qd + TET 500 mg qd + MTZ 500 mg tid, 14d LBMT: LPZ 30 mg bid + CBS 300 mg qd + TET 500 mg qd + MTZ 500 mg tid, 14d	ITT: 80.0% vs. 77.4% PP: 90.2% vs. 82.4%	39.1% vs. 43.4%	UBT
Cho et al. [74]	2024	South Korea	Retrospective cohort study	306	TBAC: TPZ 50 mg bid + BPC 300 mg bid + AMX 1 g bid + CLR 500 mg bid, 14d TAC: TPZ 50 mg bid + AMX 1 g bid + CLR 500 mg bid, 14d	ITT: 82.9% vs. 71.8% PP: 95.8% vs. 87.5%	29.2% vs. 27.3%	UBT
Kwon et al. [75]	2023	South Korea	Single-arm trial	84	TACM: TPZ 50 mg bid + AMX 1 g bid + CLR 500 mg bid + MTZ 500 mg bid, 10d	ITT: 90.5% PP: 96.2%	51.2%	UBT
Tan et al. [76]	2024	China	Randomized controlled trial	576	KBAC: KPZ 20 mg bid + AMX 1 g bid + CLR 500 mg bid + BPC 240 mg bid, 14d EBAC: EPZ 20 mg bid + AMX 1 g bid + CLR 500 mg bid + BPC 240 mg bid, 14d	ITT: 87.80% vs. 82.52% PP: 93.49% vs. 88.24%	76.31% vs. 77.62%	UBT

VPZ, Vonoprazan; TPZ, Tegoprazan; KPZ, Keverprazan; EPZ, Esomeprazole; LPZ, Lansoprazole; AMX, Amoxicillin; MTZ, Metronidazole; CLR, Clarithromycin; TET, Tetracycline; FZD, Furazolidone; BPC, Bismuth potassium citrate; CBS, Colloidal bismuth subcitrate; ITT, Intention-to-treat; PP, Per-protocol; UBT, Urea breath test; qd, Quaque die; bid, Bis in die; tid, Ter in die.

## P-CAB-based salvage therapy

The failure rate of *H. pylori* eradication treatment should not be underestimated, as it involves various complex factors such as bacterial resistance, inappropriate drug selection, poor patient compliance, and excessive gastric acid secretion. Among these, bacterial resistance is particularly prominent, especially in patients with a history of repeated treatment failures. The emergence of highly resistant strains poses significant challenges for salvage therapy. Additionally, we have noted that using PPIs before eradicating *H. pylori* can reduce the effectiveness of the treatment. The reason may be that PPIs alter the bacterial distribution (decreasing in the antrum while increasing in the corpus) [77,78]. It may lead to the bacteria potentially evading the drug or developing resistance. Although the PPI-based quadruple therapy is recommended as rescue treatment for *H. pylori* infection, its clinical efficacy has not met expectations, especially in patients with two or more previous treatment failures. Therefore, P-CAB-based regimens may offer a new treatment option.

### VPZ-based salvage therapy

Previous studies have demonstrated that for patients who have failed two treatment attempts, the use of VPZ in a triple therapy significantly enhances success rates compared to esomeprazole-based regimens [79,80]. In a meta-analysis evaluating sitafloxacin-based third-line eradication regimens, researchers systematically compared the efficacy and safety of different acid suppressants (PPI vs. VPZ). Subgroup analysis revealed that the 7-day VPZ–sitafloxacin–amoxicillin triple therapy was significantly superior to the PPI-based triple regimen (*p* < 0.001). Safety analysis indicated that diarrhea was the primary adverse reaction (24.4%), with no serious adverse events reported [81]. In conclusion, the 7-day VPZ–sitafloxacin–amoxicillin regimen represents an effective option for third-line *H. pylori* eradication therapy in Japan. However, future studies should explore whether extending the treatment duration of VPZ-based triple therapy could further improve eradication rates, as well as the validation of sitafloxacin’s therapeutic efficacy in non-Japanese populations. Additionally, safety concerns regarding fluoroquinolones must be considered, and sitafloxacin should be administered only after confirming *H. pylori* susceptibility to the drug. A meta-analysis focusing on VPZ for *H. pylori* eradication incorporated 16 observational studies from Japan, involving 6,664 patients. This analysis, employing both ITT and PP methods, compared the efficacy and safety of VPZ and PPI in eradicating *H. pylori*. The results indicated a significantly higher eradication success rate in the VPZ group than the PPI group for second-line treatment, at 91% and 88% respectively (*p* < 0.01) [82]. Furthermore, a retrospective study investigating the efficacy of a VPZ-based triple therapy in second-line treatment. The study revealed a rate of 98.0%, with good patient tolerance [61]. However, given the relatively small number of patients receiving second-line treatment, large-scale studies are needed to further validate its effectiveness and safety. A retrospective study conducted at Peking University explored the potential of VA dual therapy in the salvage treatment of *H. pylori* infection. The study enrolled 186 patients who had experienced at least one previous eradication failure, with an average of 2.1 failures. After a 14-day course of VA dual therapy, the results were encouraging, with an eradication rate of 92.5% and minimal side effects [83]. This outcome suggests that VA dual therapy is not only highly effective but also safe, offering new hope for patients who have repeatedly failed treatment. Surprisingly, a recent study revealed that the dual therapy of VPZ combined with tetracycline achieved a satisfactory eradication rate of 93.5% in patients who had previously failed amoxicillin-containing treatments for *H. pylori* infection [54]. This innovative treatment approach provides a beneficial alternative to the conventional combination with amoxicillin.

Moreover, we also noted that the addition of probiotics during VPZ therapy also provides many advantages. Probiotics offer multiple benefits, including regulating the gut microbiota, strengthening the intestinal barrier, directly inhibiting the adhesion of *H. pylori*, and alleviating inflammatory immune responses. In addition, they can reduce the side effects of antibiotics and enhance patients’ compliance with treatment. A randomized clinical study focused on the efficacy of VA dual therapy combined with Saccharomyces boulardii (*S. boulardii,* VAS) in treating *H. pylori* infection in patients with previous treatment failures and high-resistant *H. pylori* strains [84]. The results showed that VAS therapy achieved an ideal eradication rate (≥90%) with a low incidence of adverse reactions, and its efficacy was not affected by the number of previous failures. *S. boulardii* demonstrated significant effectiveness against multidrug-resistant strains, consistent with previous research [85–87]. In summary, the VAS regimen, as an important breakthrough in the treatment of *H. pylori* infection, provides an efficient and safe alternative for patients with resistant strains and those who have failed traditional therapies.

The above research findings indicate that even if the first-line treatment fails to achieve the desired outcome, a second-line treatment containing VPZ can still achieve a high eradication rate. Additionally, when necessary, the adjunct use of probiotics can further enhance therapeutic efficacy. Therefore, VPZ-containing eradication regimens are recommended as a second-line treatment strategy, aiming to significantly improve the success rate of eradication.

### TPZ-based salvage therapy

A retrospective study conducted in South Korea analyzed data from 948 patients who underwent second-line *H. pylori* eradication treatment after failing first-line therapy. Among them, 435 patients received a 7-day TPZ triple therapy, while 513 patients underwent a 14-day TPZ triple therapy. The results significantly showed that the eradication rate in the TPZ-14d treatment group was markedly superior to that in the TPZ-7d treatment group (85.1% vs. 70.5%, *p* < 0.001) [64]. Based on these findings, we speculate that in regions with high clarithromycin resistance rates, extending the duration of TPZ triple therapy to 14 days for the treatment of *H. pylori* infection may be more effective. Although current clinical research in this field is relatively limited, overall, TPZ-based salvage therapy can still serve as a potent second-line treatment option following the failure of first-line *H. pylori* eradication therapy.

In summary, in cases of *H. pylori* eradication treatment failure, the P-CAB-based dual therapy has demonstrated exceptional efficacy and safety in salvage *H. pylori* eradication treatment, earning it the title of ‘simplified salvage regimen’. Additionally, PPIs should be avoided before eradication therapy, regardless of initial or salvage treatment. Since P-CABs have a stronger acid-suppressing effect, we speculate that their long-term use might further reduce the eradication rate by affecting bacterial colonization, but this needs to be confirmed by future research.

## The impact of P-CAB therapy on the gut microbiota

During the process of eradicating *H. pylori*, antibiotics have been proven to disrupt the balance of the gut microbiota and may cause adverse events. Their use impairs the colonization ability of normal symbiotic bacteria, making it easier for exogenous pathogenic bacteria to invade the body, thereby inducing gut microbiota dysbiosis [88,89]. In addition, previous research has also suggested that changes in the composition of the gut microbiota following *H. pylori* eradication may be associated with the onset of pseudomembranous colitis and inflammatory bowel disease [90,91].

### Impact of VPZ on gut microbiota during *H. pylori* eradication

Horri et al. conducted a comparative study on the effects of VA dual therapy and VPZ-based triple therapy (VAC) on gut microbiota composition. The results showed that both α-diversity and β-diversity of the gut microbiota were significantly reduced 1–8 weeks after VAC triple therapy. However, no significant changes in α-diversity and β-diversity were observed during the same period in the VA dual therapy group [92]. In addition, another similar study compared the effects of VA dual therapy with PPI quadruple therapy on gut microbiota [38]. The VA group had a minimal impact on gut microbiota diversity and structure, and the α-diversity index of the gut microbiota in the VA dual therapy group was not significantly different from the baseline one month after treatment. In contrast, the PPI group exhibited incomplete recovery of gut microbiota structure even one month after treatment, particularly in terms of Bacteroides abundance. Compared to triple and quadruple therapies, the VA dual therapy has the least impact on the diversity and relative abundance of the gut microbiota, thereby providing a safer *H. pylori* eradication regimen.

Short-chain fatty acids (SCFA) are primarily produced in the gut through the anaerobic fiber fermentation by gut microbiota. Besides playing a crucial role in energy metabolism and supply, SCFAs are also essential for maintaining the integrity of the intestinal barrier, preventing microbial translocation, and alleviating inflammatory responses [93,94]. This study investigated the effects of different VA combination therapies on gut microbiota and SCFA levels after *H. pylori* eradication [95]. The results indicated that changes in the gut microbiota were not related to SCFA levels, and the VA dual therapy had the least impact on both gut microbiota and SCFA levels. Therefore, we believe that the VPZ-based dual therapy is a safer option for *H. pylori* eradication.

### Impact of TPZ on gut microbiota during *H. pylori* eradication

A recent randomized clinical study compared the effects of TA dual therapy versus PPI quadruple therapy on the gut microbiota [56]. The results showed that TA dual therapy significantly increased the α-diversity of the gut microbiota at 2 weeks post-treatment (*p* < 0.05) and induced notable changes in β-diversity. However, these microbial diversity levels returned to baseline 4 weeks after treatment discontinuation. In stark contrast, PPI quadruple therapy still exhibited a declining trend in α-diversity even at 4 weeks post-treatment. Further analysis revealed that the impact of TA dual therapy on the gut microbiota was transient and reversible, primarily characterized by a short-term increase in diversity and fluctuations in certain bacterial genera during treatment, with full recovery observed within 4 weeks after cessation. Compared to traditional multi-antibiotic regimens, TA dual therapy demonstrated superior safety in maintaining gut microbial balance, suggesting a milder disruption to the gut ecosystem. Interestingly, Son et al. explored the effects of TPZ on gut microbiota and intestinal barrier integrity in a mouse model. Their findings indicated that TPZ enhances intestinal barrier function, promotes the growth of beneficial bacteria, suppresses pathogenic bacteria, and reduces inflammation, thereby effectively preventing colitis [96].

The above findings suggest that a dual therapy regimen containing P-CAB and only one antibiotic can effectively minimize adverse effects on the gut microbiota, making it a potentially more suitable option for maintaining long-term microbial ecological health.

## Conclusion

P-CAB, a novel acid suppressant, reversibly blocks H^+^/K^+^-ATPase for sustained acid inhibition, overcoming PPI limitations. P-CAB regimens offer high eradication rates, good safety, and work against resistance. Dual therapy simplifies antibiotics, reducing side effects and minimally disrupting gut flora, which is crucial for patients needing repeated treatment or having fragile microbiomes.

Although P-CABs show great potential for *H. pylori* eradication, further research is needed to expand their clinical evidence: (1) Expand RCT studies for TPZ/KPZ; (2) Conduct large-sample, multicenter clinical research to further validate the efficacy and safety of P-CAB combined with other antibiotics (such as tetracycline) in treating penicillin-allergic patients, and explore its efficacy and safety in different populations (non-penicillin-allergic patients or non-penicillin-allergic individuals who have previously taken amoxicillin for other conditions or have previously failed *H. pylori* eradication treatment involving amoxicillin); (3) Continue to investigate the long-term impact of different P-CAB therapies on the gut microbiota and their role in reducing antibiotic resistance. Future large-scale studies are needed to confirm P-CAB’s effectiveness against *H. pylori*, aiming to increase eradication rates, reduce gastric cancer risk, and improve patient well-being.

## Data Availability

Data sharing is not applicable to this article as no new data were created or analyzed in this study.
